# Prevalence of human papillomavirus genotypes among women with cervical cancer in Ghana

**DOI:** 10.1186/s13027-016-0050-4

**Published:** 2016-01-26

**Authors:** A. K. Awua, S. T. Sackey, Y. D. Osei, R. H. Asmah, E. K. Wiredu

**Affiliations:** Department of Biochemistry, Cell and Molecular Biology, University of Ghana, Legon, Accra Ghana; Cellular and Clinical Research Centre, Radiological and Medical Science Research Institute, Ghana Atomic Energy Commission, Accra, Ghana; Department of Medical Laboratory Sciences, School of Biomedical and Allied Health Science, College of Health Sciences, University of Ghana, Korle-Bu, Accra, Ghana; Department of Pathology, School of Biomedical and Allied Health Science, College of Health Sciences, University of Ghana, Korle-Bu, Accra, Ghana; University of Health and Allied Sciences, Ho, Ghana

**Keywords:** Nested PCR, Multiplex PCR, Human Papillomavirus, Cervical cancer, Paraffin-embedded tissues, Ghana

## Abstract

**Background:**

Human Papillomavirus (HPV) infections have been shown to be a necessary risk factor for the development of cervical cancer. However, HPV genotype distribution varies geographically, both in type and relative prevalence. In order to ensure a successful introduction of available vaccines, there is the need to identify pre-vaccination HPV genotype prevalence in Ghana and the extent of single and multiple-infections.

**Methods:**

Paraffin-embedded cervical tissues of 256 confirmed cervical cancer cases diagnosed at the Korle-Bu Teaching Hospital during the period January 2004 to December 2006 were selected after hematoxylin and eosin staining and confirmation. Following a heat-proteinase K-based tissue lysis, HPV was detected and typed by a nested-multiplex PCR assay using an E6/E7 consensus primer and type-specific primers.

**Results:**

Of the 256 cases, 230 (89.8 %, 95 % CI 85.7–93.4 %) were positive for HPV DNA. HPV18 (47.4 %), HPV59 (42.2 %), HPV45 (37.4 %) and HPV16 (9.0 %) were the four common HPV genotypes detected. A total of 110 (47.8 %) of the 230 HPV DNA positive tissues, were infected by a single HPV genotype while the other 120 (52.2 %) were infected by multiple HPV genotypes. A significant association was determined between each of the following HPV genotypes and multiple-infection; HPV18 (OR = 6.97; 95 % CI, *3.89–12.50)*, HPV59 (OR = 9.56; 95 % CI, *5.57–20.02*) and HPV45 (OR = 1.94; 95 % CI, *1.12–3.35*).

**Conclusion:**

The prevalence of the following high risk HPV genotypes (HPV18, HPV59, HPV45) were relatively high among the cases of cervical cancers reported at this hospital in Ghana during the study period. Additionally, there was a high frequency of HPV multiple-infections among these cases.

## Background

The West African region was estimated to bear the second highest burden of cervical cancer, with a mortality to incidence ratio of 81.2 % and cervical cancer was estimated to be the most frequent cancer among women in Ghana as at the time of this study [1]. The age standardised ratio (ASR) of 39.5 per 100,000 women [1]. An earlier study at the Korle-Bu Teaching Hospital also reported that 58.3 % of gynaecological cancers seen at the hospital in the year 2000 were cases of cervical cancer [2]. At the same hospital, a 10 year (1991–2000) study of cancer mortality pattern revealed that cervical cancer was one of the four leading causes of cancer deaths (Age Standardized Cancer Ratio of 8.74 %) in females [3].

A number of studies have shown that persistent infections with certain human Papillomavirus (HPV) genotypes known as high risk HPV type (HPV-16, -18, -31, -35, -39, -45, -51, -52, -56, -58, -59 and -68) are necessary in the aetiology of cervical cancer. These high risk HPV genotypes have been shown to be associated with greater than 99.0 % of all cervical cancer cases [4–7]. Of these high risk HPV genotypes HPV16 and HPV18 are the two prevalent HPV types in cervical carcinoma globally and are associated with approximately 60.0 % and 10.0 % of cervical cancer cases respectively [8–10]. However, epidemiological studies have shown a global geographical variation in the type specific and relative HPV prevalence among different populations; these range between 2 and 44 % [11]. Fortunately, there are indications of high efficacy of available vaccines against HPV infections and a greater possibility of preventing cervical cancer. However, it not clear how the population specific HPV genotype distribution, extent of multiple-infections and the HPV types involved in these multiple-infections will change following a successful implementation of these efficacious vaccines. Evidence is building on the occurrence of cross protection against non-vaccine HPVs and the possibility of the occurrence of HPV genotype replacements among vaccinated populations. In order to determine the occurrence of such changes, knowledge of the pre- and post-vaccination HPV genotype distribution is very important.

However, empirical data on the overall and HPV genotype specific prevalence in cases of cervical cancers in Ghana were very few at the time of this study. Additionally, and in order to evaluate the potential benefits of an introduction of HPV vaccination in Ghana, this study was designed to detect, genotype and determine the extent of multiple HPV infection using archival formalin-fixed paraffin-embedded cervical tumour specimen collected from the Pathology Department of Korle-Bu Teaching Hospital during the years 2004–2006. For the detection of HPV, a nested-multiplex PCR method that had been shown to be very sensitive and specific, with detection rates of between 91.8 % for cervical intraepithelial neoplasia (CIN I) and 99.3 % for CIN III was used [12].

## Method

### Sample collection and DNA extraction

The Korle-Bu Teaching Hospital is one of the tertiary referral centre for persons or specimens with any form of suspected malignancy in Ghana. Paraffin-embedded formalin-fixed cervical tissues of women who were referred to the Pathology Department of the Korle-Bu Teaching Hospital, Accra, between January 2004 and December 2006 and diagnosed histologically with high grade precursor lesion (CIN III, high grade squamous Intraepithelial lesion (HSIL), or carcinoma in situ) or cervical cancer (adenocarcinoma, adenosquamous carcinoma or any form of squamous cell carcinoma), were selected for this study. Cases diagnosed as adenocarcinoma and adenosquamous were grouped together as invasive adenocarcinoma (IAC), any form of squamous cell carcinoma were grouped as invasive squamous cell carcinoma (ISCC) and all forms of carcinoma in situ were grouped as carcinoma in situ (CIS).

Each tissue block was sectioned to obtain three 9-μm sections, each with a separate and sterile microtone blade. The middle section was stored in a 2 mL sterile microfuge tube for DNA extraction. The first and third sections were stained with hematoxylin and eosin (H/E) and examined for cancerous tissues by 2 Consultant Pathologist. The presence of cancerous tissue in the stained sections confirmed that the middle section used for DNA extraction actually contained cancer tissue. 256 samples, consisting of 10 cases of carcinoma in situ (CIS), 19 cases of invasive adenocarcinoma (IAC) and 227 cases of invasive squamous cell carcinoma (ISCC) were found to have contain cancerous tissues and so were used for subsequent analysis.

DNA was extracted from the 256 samples, as described by Dabic’ et al., [13] with slight modifications. In brief, 10 μm section was incubated in 250 μL of extraction buffer (consisting of 1 mg/mL Proteinase K in 50 mM Tris-HCl pH 8, 1 mM EDTA and 0.5 % Tween-20) for 16 h at 56^O^C. The proteinase K was thereafter inactivated by heating at 100^O^C for 5 min. The tubes were allowed to cool to room temperature after which the tubes were centrifuged at 5000xg for 5 min. The tissue lysates were transferred for storage at -20 °C as aliquots of 70 μL. Positive and negative controls (previously confirmed cancerous and non-cancerous cervical tissues respectively), were used for the DNA extraction.

### Detection and genotyping of HPV by nested-multiplex PCR

The method and primer sets used for the nested-multiplex PCR were as described by Soltar et al., [14] with slight modification. Its sensitivity was as higher as that of the MY/GP primer assay (10^2^–10^1^ viral copy detection) [14] and an initial adoption of this method in our laboratory showed it was effective for the purpose of genotyping the high risk HPVs [15]. This assay targets the detection of 18 HPV genotypes (6/11, 16, 18, 31, 33, 35, 39, 42, 43, 44, 45, 51, 52, 56, 58, 59, 66 and 68) with a sensitivity comparable to that of the MY/GP assay but higher than those of the MY09-MY11 and GP5 + -GP6+ assays which amplifies the L1 region of the HPV genome. Briefly, HPV DNA in the lysate was amplified using a single consensus forward primer, GP-E6-3 F [GGGWG KKACT GAAAT CGGT], and two consensus reverse primers GP-E7-5B [CTGAG CTGTC ARNTA ATTGC TCA] and GP-E7-6B – [TCCTC TGAGT YGYCT AATTG CTC]. The four second round primer-cocktail-sets, as shown in Table 1, were used for the genotyping. Both first and second round PCRs were performed in a final volume of 25 μL and each PCR mixture contained 50 mM KCl, 9 mM Tris-HCl (pH 9.0), 1 % Triton-X100, 2.0 mM MgCl_2_, 0.2 mM of each dNTP, 320 nM of each of the primers and 1.25 U of Taq polymerase. The amplifications were carried out using a thermal cycler (Applied Biosystems 1720) with the following parameters:

**Table 1:** Sequences of synthetic oligonucleotide primers used for HPV genotyping

Primer cocktail	HPV genotype primer	Size of amplicon (bp)	Sequence 5′to 3′)
Consensus primers	GP-E6-3 F		GGGWG KKACT GAAAT CGGT
	GP-E7-5B		CTGAG CTGTC ARNTA ATTGC TCA
	GP-E7-6B		TCCTC TGAGT YGYCT AATTG CTC
Cocktail 1	HPV16f	457	CACAG TTATG CACAG AGCTGC
	HPV16r		CATAT ATTCA TGCAA TGTAG GTGTA
	HPV18f	323	CACTT CACTG CAAGA CATAG A
	HPV18r		GTTGT GAAAT CGTC GTTTT TCA
	HPV31f	263	GAAAT TGCAT GAACT AAGCT CG
	HPV31r		CACAT ATACC TTTGT TTGTC AA
	HPV59f	215	CAAAG GGGAA CTGCA AGAAA G
	HPV59r		TATAA CAGCG TATCA GCACC
	HPV45f	151	GTGGA AAAGT GCATT ACAGG
	HPV45r		ACCTC TGTGC GTTCC AATGT
Cocktail 2	HPV33f	398	ACTAT ACACAACATT GAACT A
	HPV33r		GTTTT TACAC GTCAC AGTGC A
	HPV6_11f	334	TGCAA GAATG CACTG ACCAC
	HPV6_11r		TGCAT GTTGT CCAGC AGTGT
	HPV58f	274	GTAAA GTGTG CTTAC GATTG C
	HPV58r		GTTGT TACAG GTTAC ACTTG T
	HPV52f	229	TAAGG CTGCA GTGTG TGCAG
	HPV52r		CTAA TAGTT ATTTCA CTTAA TGGT
	HPV56f	181	GTGTG CAGAG TATGT TTATT G
	HPV56r		TTTCT GTCAC AATGC AATTG C
Cocktail 3	HPV35f	358	CAACG AGGTA GAAGA AAGCA TC
	HPV35r		CCGAC CTGTC CACCG TCCAC CG
	HPV42f	277	CCCAA AGTAG TGGTC CCAGT TA
	HPV42r		GATCT TTCGT AGTGT CGCAG TG
	HPV43f	219	GCATA ATGTC TGCAC GTAGC TG
	HPV43r		CATGAAACTG TAGAC AGGCC AAG
	HPV44f	163	TAAAC AGTTA TATGT AGTGT ACCG
	HPV44r		TATCA GCACG TCCAG AATTG AC
Cocktail 4	HPV68f	333	GCAGAAGGCA ACTAC AACGG
	HPV68r		GTTTA CTGGT CCAGC AGTGG
	HPV39f	280	GACGACCACT ACAGC AAACC
	HPV39r		TTATG AAATC TTCGT TTGCT
	HPV51f	223	GAGTA TAGAC GTTAT AGCAG G
	HPV51r		TTTCG TTACG TTGTC GTGTA CG
	HPV66f	172	TTCAG TGTAT GGGGC AACAT
	HPV66r		AAACA TGACC CGGTC CATGC

*F*, *f* forward, *r* reverse, *B* back

Single letter code: W = A/T; K = G/T; R = A/G; Y = C/T; N = A/C/G/T; X = unknown. (Adapted from Soltar et al., [14])

For the first round PCR, using 5.0 μL or 1 μL of lysate, an initial denaturation at 94 °C for 4 min was followed by 40 cycles of denaturation at 94 °C for 1 min, annealing at 40 °C for 2 min and an extension at 72 °C for 2 min. A single final extension at 72 °C for 9 min was performed before a soaking step at 4 °C. For the second round PCR, 2.0 μL of the first round PCR product were used as the template DNA with each of the four cocktails of type specific primers for genotyping (Table 1). The cycling parameters were as follows: an initial denaturation at 94 °C for 4 min was followed by 35 cycles of denaturation at 94 °C for 30 s, annealing at 56 °C for 30 s, and an extension at 72 °C for 45 s. This was followed by a single final extension at 72 °C of 4 min, before a soaking step at 4 °C. For each round of PCR reaction, HPV16 or/and HPV18 genome in plasmid DNA as well as a sample positive for HPV used in the optimisation of PCR were used as positive controls while nuclease free water and DNA extracts form the non-cancerous tissues was used as a negative control. A sample each, found to be HPV negative or positive, were subsequently used as additional negative and positive control, respectively.

Following the second round PCR, 8.0 μL of the products were resolved on a 2 % agarose gel stained with 0.001 mg/mL ethidium bromide. The electrophoresis was carried out in 1X Tris Acetate EDTA (TAE) buffer at 100 V for 1 h and the gel photographed over a UV trans-illuminator. The molecular weights of the resolved PCR products were used to determine the genotypes of HPV detected for each sample according to the expected weight for each primer (Table 1).

### Statistical analysis

Data were managed and analysed with the Epi Info statistical software, version 3.3 (CDC, Atlanta, USA). The number and proportion of cases of carcinoma in situ (CIS), invasive adenocarcinoma (IAC) and invasive squamous cell carcinoma (ISCC) were determined. Furthermore, the number and proportions of detected HPV genotypes, stratified by mode of infection (overall, single and multiple infections), type of cervical cancer case and age of the women were determined and presented in frequency tables. The association between each HPV genotype and multiple infection status was determined by an analysis of odds ratio.

## Results

**Fig. 1:**
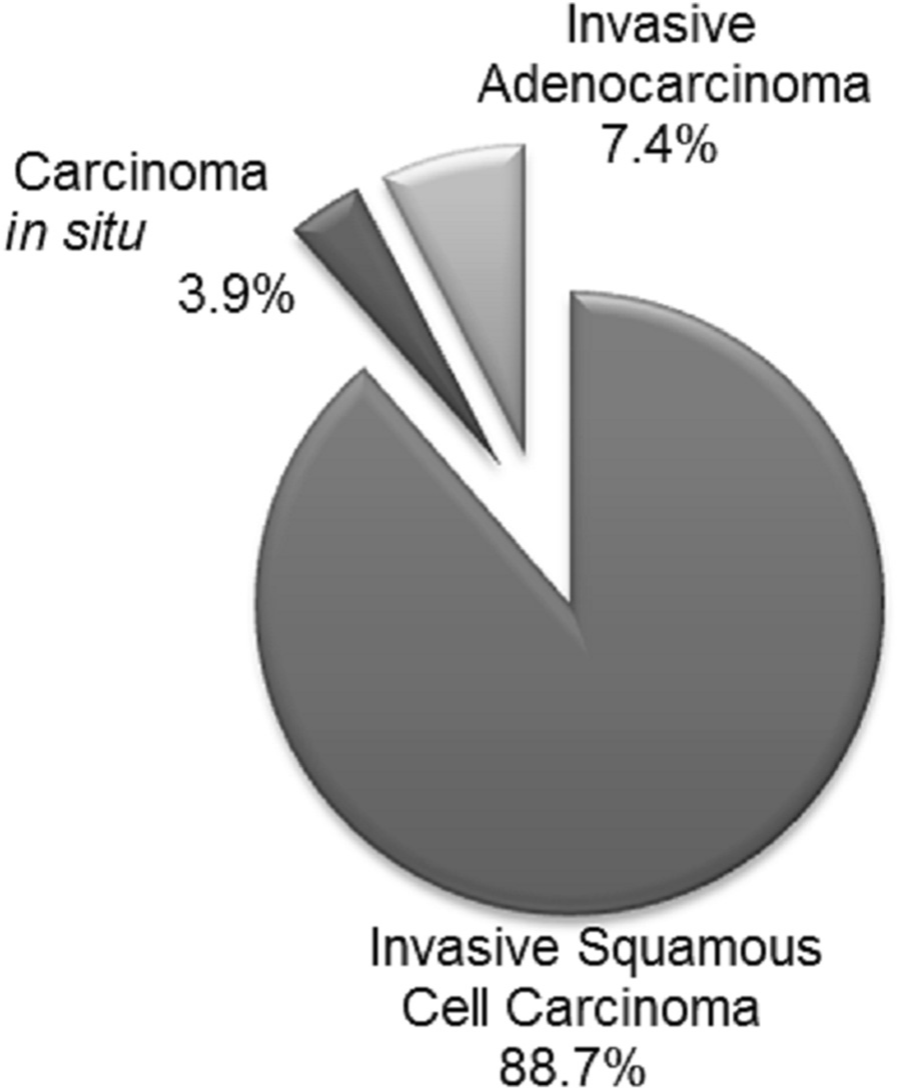
Distribution of diagnosed cervical cancer cases

After the hematoxylin and eosin staining of the second 10 μm section of each of the 342 paraffin embedded formalin fixed cervical cancerous tissue blocks, 256 were determined to have been adequate for PCR testing; These consisted of 10 (3.9 %) cases of carcinoma in situ (CIS), 19 (7.4 %) cases of invasive adenocarcinoma (IAC) and 227 (88.7 %) cases of invasive squamous cell carcinoma (ISCC), as shown in Fig. 1.

**Fig. 2:**
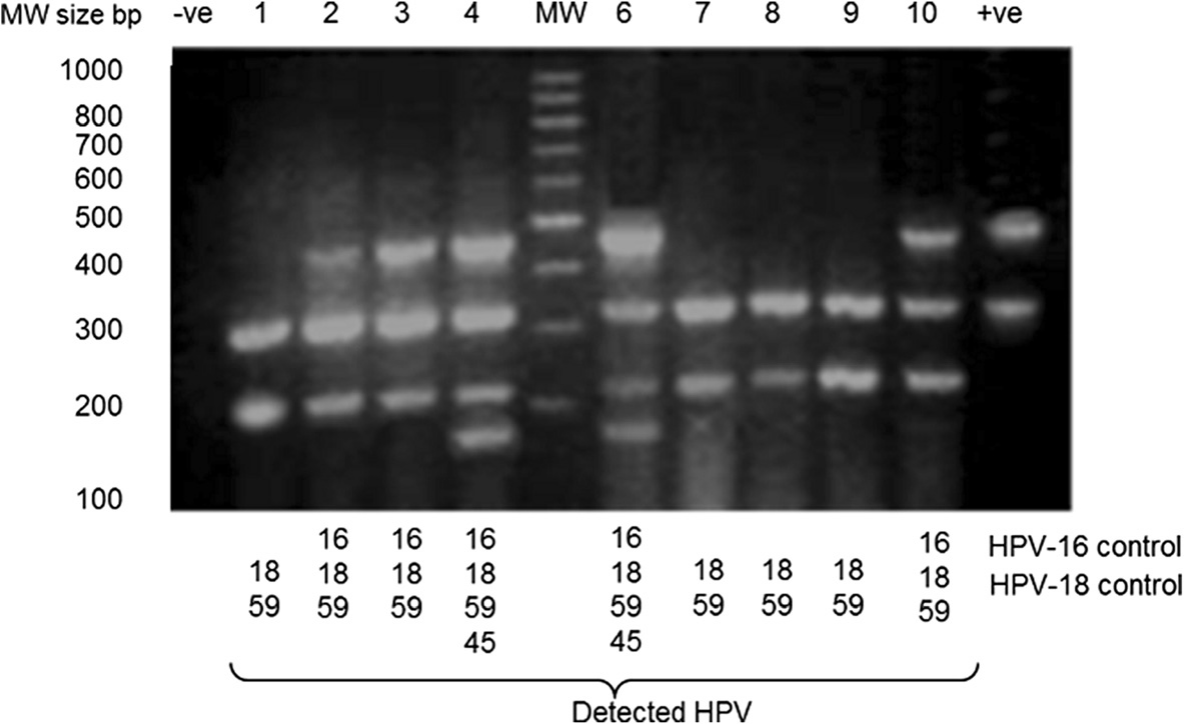
Detected type specific HPV DNA in multiple infections. Round 2 amplification products were resolved at 100 V for 1 h on a 2 % agarose gel stained with 0.001 mg/mL of ethidium bromide and photographed under UV illumination. The HPVs were identified based on the molecular weight of the amplification products for each are shown below the gel. Lane -ve ⇒ HPV negative control; Lane MW ⇒ Bands of the 100 base pair molecular weight maker; Lane + ve ⇒ HPV16 and HPV18 DNA positive control

HPV-specific DNA was detected in 230 samples (Fig. 2), which is a prevalence of 89.8 % (95 % CI 85.7–93.4 %). These positive samples were distributed among the diagnosis categories as follows, 90.0 % (9 of 10) Carcinoma in situ (CIS) cases, 89.5 % (17 of 19) invasive adenocarcinoma (IAC) cases and 89.9 % (204 of 227) invasive squamous cell carcinoma (ISCC). The four commonly detected (overall prevalence) HPVs were HPV18 (47.4 %), HPV59 (42.2 %), HPV45 (37.4 %) and HPV16 (10.0 %) (Table 2). Single infections were observed among 110 (47.8 %) of the 230 HPV DNA positive cases and the same four HPV genotypes were the commonest single infecting genotypes but in a different order. This was as follows, HPV45 (29.4 %), HPV18 (23.95 %), HPV59 (15.6 %) and HPV16 (9.2 %). Eight of the single infections were solely of low-risk HPV types (Table 2), these were; 4 ISCC cases with HPV44, 1 CIS and 1 ISCC case with HPV6_11 and 2 ISCC cases with HPV42. Multiple-infections occurred in 120 (52.2 %) of the 230 DNA positive cases. Double infections were observed in 90 (39.1 %), triple infection in 28 (12.0 %) and quadruple infections in 5 (2.2 %) of the 230 HPV positive cases (Table 3). Double infections involving HPV18 were the commonest. Those with HPV18 and HPV59 occurred in 30 (13.0 %) cases, those with HPV18 and HPV16 occurred in 3 (1.3 %) and those with HPV18 and HPV45 occurred in 2 (0.9 %) cases. Furthermore, the commonest triple HPV infections were detected in 17 (7.3 %) cases and these involved HPV-18, -45 and -59. All the other 9 triple infections were each detected once while all the 5 quadruple infection involved HPV-16, -18, -45 and -59 (Table 3).

**Table 2:** Distribution of specific HPV genotypes detected in cancerous cervical tissues, occurring either as single infections or alongside others in multiple-infections

HPV type	Overall, (%)^a^ (*N* = 230)	Multiple-infections (%)^b^ (*N* = 120)	Single infections (%)^c^ (*N* = 110)	Odds ratio (OR)	*95 % CI*
18	109 (47.4)	83 (68.6)	26 (23.9)	6.97^d^	*3.89–12.50*
59	97 (42.2)	80 (66.1)	17 (15.6)	10.56^d^	*5.57–20.02*
45	86 (37.4)	54 (44.6)	32 (29.4)	1.94^d^	*1.12–3.35*
16	23 (10.0)	13 (10.7)	10 (9.2)	1.19	*0.50–2.84*
31	20 (8.7)	14 (11.6)	6 (5.5)	2.50	*0.83–6.07*
56	14 (6.1)	9 (7.4)	5 (4.6)	1.67	*0.54–5.15*
6_11	10 (4.3)	6 (5.0)	2 (1.8)	1.40	*0.37–1.99*
35	8 (3.5)	6 (5.0)	2 (1.8)	2.79	*0.55–14.13*
42	7 (3.0)	4 (3.3)	2 (1.8)	1.21	*0.26–5.52*
44	7 (3.0)	3 (2.5)	4 (3.7)	0.66	*0.15–3.05*
58	5 (2.2)	3 (2.5)	2 (1.8)	1.36	*0.22–8.30*
33	3 (1.3)	3 (2.5)	0 (0.0)	UD	*UD*
66	2 (0.9)	1 (0.8)	1 (0.9)	0.90	*0.06–14.57*
43	1 (0.4)	1 (0.8)	0 (0.0)	UD	*UD*
51	1 (0.4)	0 (0.0)	1 (0.9)	UD	*UD*

UD undeterminable, as a result of less than five detections of each HPV for each state of infection

OR = Measures the odds of the association between being positive for each type HPV and the state of multiple-infection

^a^percentage of total infections

^b^percentage of total multiple-infections

^c^percentage of total single infections

^d^Significantly associated with multiple-infections because odds ratio is not = 1.0 and a 95 % CI does not overlapping 1.0

**Table 3:** Distribution of multiple- and double infections involving HPV-18 and the other most detected HPV genotypes

Multiple-infection	Number (% of total HPV infections)
Double infections	90 (39.1)
HPV-18 and HPV-59	30
HPV 18 and HPV 31	4
HPV-18 and HPV-16	3
HPV-18 and HPV 45	2
Triple infections	25 (10.8)
HPV-18,-45 and -59	17
HPV-18,-31 and 6_11	1
HPV-18, -35 and -42	1
HPV-18, -35 and -58	1
HPV-18, -42 and -6_11	1
HPV -18, -45 and -56	1
HPV -18, -59 and -6_11	1
HPV -16, -18 and -31	1
HPV-16, -18 and -59	1
HPV-16, -42 and -66	1
HPV-31, -45 and 6_11	1
HPV-45, -56 and 6_11	1
Quadruple infections	5 (2.2)
HPV-16, -18, -45 and -59	5

An age stratified distributions of the data showed that the age groups 40–59 years and 60–79 years carried the highest burden of each diagnosis categories of cervical cancer, overall HPV infection positivity, single and multiple HPV infections (Table 4). Specifically, 63.2, 60.0 and 44.5 % of the IAC, CIS and ISSC cases were observed among the age range of 40–59 years. Furthermore, 46.5 % of all HPV infections, 51.4 % of single infections and 42.4 % of multiple infections (representing the highest proportion in category) were detected among cases of this age range.

**Table 4:** Age stratified distribution of histologic type and HPV infection among the cervical cancer cases

Age group (years)	Histological type, *n* (%)			HPV status, *n* (%)		Type of Infection, *n* (%)	
	CIS	IAC	ISCC	HPV (−)	HPV (+)	Multiple	Single
20–39	1 (10.0)	1 (5.3)	17 (7.5)	4 (15.4)	15 (6.5)	7 (5.8)	8 (7.2)
40–59^a^	6 (60.0)	12 (63.2)	101 (44.5)	12 (46.2)	107 (46.5)	51 (42.5)	56 (50.9)
60–79	3 (30.0)	5 (26.3)	89 (39.2)	10 (38.5)	88 (38.3)	50 (41.6)	38 (34.5)
>79	0 (0.0)	0 (0.0)	18 (7.9)	0 (0.0)	18 (7.8)	10 (8.3)	8 (7.3)
-	0 (0.0)	0 (0.0)	2 (0.9)	0 (0.0)	2 (0.9)	2 (1.6)	0 (0.0)
Total	10	19	227	26	230	120	110

^a^Age group with significantly higher proportions of cases and HPV infection

Specific genotype distributions among the diagnosis categories were presented in Table 5. HPV16 was observed in 3 (33.3 %) of the 9 CIS cases, 3 (15.8 %) of the 19 IAC cases and 17 (7.5 %) of the 227 ISCC cases. HPV18 was observed in 3 (33.3 %) of the 9 CIS cases, 7 (36.8 %) of the 19 IAC cases and 99 (43.6 %) of the 227 ISCC cases. HPV 6_11was observed in 2 (22.2 %) of the 9 CIS cases, 8 of the 227 ISCC cases but was not observed in IAC cases. Furthermore, Table 6 shows the age distribution of the commonly detected HPV genotype. The age groups 60–79 years harbored a high proportion of HPV-18, 59 and 45 although the age group 40–59 years harboured the highest overall HPV infection (Table 4).

**Table 5:** Distribution of the common HPV genotypes among the types of cancers

HPV genotype	Number (%)		
	CIS	IAC	ISCC
18	3 (30.0)	7 (36.8)	99 (43.6)
59	4 (40.0)	8 (42.1)	85 (37.4)
16	3 (30.0)	3 (15.8)	17 (7.5)
45	0 (0.0)	7 (36.8)	79 (34.8)
35	1 (9.0)	0 (0.0)	7 (3.1)
6_11	2 (20.0)	0 (0.0)	8 (3.5)
56	0 (0.0)	1 (5.3)	y5.7)
Total tested	10	19	227

% are of the total of each type of cancer (because of multiple-infections, the percentage may add up to more than 100)

**Table 6:** Distribution of the most frequent HPV genotype infection among cervical cancer patient according to age

HPV type	Number (%) of cancer patients			
	20–39 years (*n* = 15)	40–59 years (*n* = 107)	60–79 years (*n* = 88)	>79 years (*n* = 18)
18	7 (46.7)	48 (44.9)	47 (53.4)	7 (38.8)
59	6 (40.0)	40 (37.4)	42 (47.7)	9 (50.0)
16	1 (6.6)	13 (12.1)	7 (8.0)	2 (11.1)
45	6 (40.0)	36 (33.6)	37 (42.0)	7 (38.8)
35	1 (6.6)	3 (2.8)	3 (3.4)	1 (5.5)
42	0	4 (3.7)	2 (2.3)	1 (5.3)
6_11	0	6 (5.6)	4 (4.5)	0 (0.0)
44	0	2 (1.9)	3 (3.4)	2 (11.0)

The number HPV risk types may add up to more than the total number of cancer patients for each age group due to multiple-infection of some patients or may be less than reported in Table 2 because some participants’ age was not available. % are of the total number of HPV positive cases (presented as n) in each age range

## Discussion

As shown in Fig. 1, the observed distribution of cervical cancer cases was in line with global observations reported for the study period [16] and the age group with the highest burden of cervical cancer in this study was consistent with global data on cervical cancer (Table 4). Similarly, the prevalence of HPV DNA (89.8 %, 95 % CI 85.7–93.4 %) among these cases of cervical cancer, which was based on the amplification of the viral *E6* and *E7* oncogenes, was comparable to that (89.4 %) reported by a study of cervical tumour samples in the neighbouring Cote d’Ivoire [17]. Additionally, a comparable HPV prevalence of 93.9 % was reported among cases diagnosed as CIN II or higher in the study from which the HPV detection and genotyping methods were adapted [14]. However, in an earlier study of 50 similarly processed samples collected between January and December 2003 at the same hospital and using the same HPV detection methods, an HPV positivity of 98.0 % was reported [15]. Additionally, data reported for Ghana in a multi-centre study, which used biopsy samples collected between October 2007 and March 2010 and a different HPV detection and genotyping methods, indicated an overall HPV prevalence of 93.9 % [18]. In respect of the genotype specific prevalence (Table 2), HPV18 was the commonest genotype detected in this study as was the case the earlier study in Ghana [15], however, HPV16 was the commonest for the Ghanaian data reported in the multi-centre study [18].

Although these overall HPV prevalence were within the expected range of 90–100 % [19] and that there are reports of within country variation in HPV genotype specific prevalence [11], the differences between these studies may have been influenced by the following facts. The first is the difference in the type of specimen used in these studies. Specifically, this and the study by Attoh et al., [15] used archived formalin-fixed paraffin-embedded tissue blocks while the study by Denny et al., [18] used freshly collected biopsy samples. Also, the processing of the archived tissue blocks used in this studyand that by Attoh et al., [15] were not standardized and therefore different levels of inhibitors may have been present in the samples and in extracted DNA. The second was that the cases in these studies were received from different locations across the country and therefore the differences in prevalence may be a reflection of the inter-country variations. Thirdly, the variations in the times of samples collection, without overlaps, (2003, 2004–2006 and 2007–2010) and the relatively small number (*n* = 50) of samples used in the study by Attoh et al., [15] may have contributed to the differences in the data of these studies.

Inrespect of the genotype specific prevalence, the high prevalence of HPV59 and its frequency in multiple infections in this study remains unclear. However, the differences in these three studies most probably are a reflection of the variability in the HPV prevalence in the Ghanaian population and therefore there is the need for a well-controlled randomized population based HPV prevalence study in Ghana. On the other hand, a comparison of these HPV distributions with those of Ghana’s neighbouring populations strongly supports the existence of geographical difference in the prevalence of HPV genotypes and the possibility that HPV16 may not be the most prevalent genotype in these African countries. For instance, a study in Benin reported HPV59 (24.6 %), HPV35 (22.5 %), HPV16 (17.6 %), and HPV18 (14.8 %) as the common HPV genotypes detected [15]. Also, a study in Cote d’Ivoire reported HPV16 (45.0 %), HPV18 (21.0 %), HPV45 (9.0 %), HPV35 (8.0 %), and HPV31 (3.0 %) as the common genotypes [17] while in a study in Burkina Faso, HPV52 (14.7 %), HPV35 (9.4 %), HPV58 (9.4 %), and HPV51 (8.6 %) were the common genotypes [20]. Furthermore, study form other regions in and outside Africa confirm the assertion that although HPV16 and HPV18 are the commonest HPVs in cervical cancers globally, they are not always the two commonest HPVs in every country. For instance, in Tanzania, HPV16 and HPV58 were the first two prevalent genotypes while HPV18 was the fifth [21]; In Mozambique, HPV35 was the most prevalent HPV genotype while no HPV18 genotype was detected among women diagnosed with HSIL or carcinoma [22]. Liaw et al., [23] reported HPV52 and HPV58 as the most prevalent type in parts of China.

Interestingly, HPV18, HPV59 and HPV45, which are of the same phylogenetic family [7], as expected were the common genotypes in adenocarcinoma (Table 5). On the other hand, HPV16 and its phylogenetic related family members, HPV31 and HPV35 were respectively the fourth, fifth and eighth prevalent HPV in this study. Furthermore, most of the multiple-infections observed in this study involved HPV18 and HPV59. These suggest that a phylogenetic dependency in the colonization of cervical epithelium might contribute to HPV prevalence, as was observed by Conesa-Zamora et al., [24] for HPV18 and HPV45. Therefore, these may suggest a phylogenetic related HPV prevalence in Ghana, although the bases for such specificities are still not clear.

Although, the frequency of multiple-infections varies with the type of HPV detection method used [25], the 52.2 % multiple-infections observed in this study as compared to that of the earlier Ghanaian study, 19.6 % [18], are discussed in light of the fact that most (about 96 %) of the cases in this study (Fig. 1) were invasive cancers (IAC and ISSC) which are known to be associated with high multiple-infections [25]. Also, data from neighbouring countries have shown similar high frequencies of multiple-infections. A 52.9 % rate of multiple-infection was observed in a study in Burkina Faso [20], while a 40.2 % rate was reported by a in Benin [19]. These may suggest a high rate of multiple-infection in the West African region. However, these may be a population specific observation as was shown by two studies in Spain, a 25.6 % rate of multiple-infection among HSIL cases in Southern Spain [24], while a 34.0 % was observed in a cohort of women in Madrid [24, 26].

Since both cross-protection of the available HPV16/18 vaccines and its clinical relevance determined with the data available for vaccine efficacy have shown additional protection against HPVs -31, -33, -45, -51, -52, -56 and -58 [27, 28], the expected impact of HPV vaccination on cervical cancers in Ghana may be further increased. Specifically, if the infection by HPV59 depends on a prior infection by HPV18 [24] since they most occurred together in this study, then a lower prevalence of both HPV18, HPV 59 and lower frequency of multiple infection may result after the introduction of the HPV16/18 vaccines.

Another finding worth commenting on was the observation that low risk HPV types (HPV-6/11, -42 and -44) were solely detected as single infections in 8 cervical cancer cases (1 CIS, 7 ISCC). These were least expected and may be misleading in suggesting a higher oncogenic potential for these low risk HPV types since low risk type HPVs are rarely observed as single infections in invasive cancers [7]. However, due to the limitation associated with DNA extract and PCR using formalin-embedded paraffin-embedded specimen (presence of inhibitors from sample fixatives), it was possible that the other multiple-infecting high risk HPV genotypes present were not detected since DNA extracts from paraffin-embedded formalin-fixed tissue samples have been reported to intermittently fail to amplify by PCR [29]. Specifically, because PCR inhibitors may have been be present at varying concentrations, the concentration of the target DNA and its quality may have been greatly reduced after tissue processing and/or the target viruses may have been heterogeneously distributed in the cancerous tissues [29–31]. The limitation of this study includes the fact that it was not powered to determine the associations between HPV genotypes and the diagnosis categories of cervical cancer. Also, the HIV statuses of the patients, which may influence HPV prevalence, were not determined.

## Conclusion

Overall, the findings of this study indicate a peculiar HPV profile for Ghana, which has important implication for the introduction of HPV vaccination and forms part of a growing body of evidence of the pre-vaccination HPV prevalence for Ghana. However, rigorous epidemiologic data and well-controlled randomized trials are needed in order to estimate the extent of protection or prevention of cervical cancer that may be achieved with the introduction of HPV vaccination in Ghana; particularly in light of the high multiple-infection prevalence observed in this study. These also underscore the need for community based screening of women for both cervical cancer and HPV infection, which should include HPV genotype analysis as part of the screening schemes for women with high-grade neoplasia and cervical cancer in Ghana. These will help throw more light on the high rate of multiple-infections, particularly those involving HPV59.
